# Effect of *in ovo* folic acid injection on hepatic *IGF2* expression and embryo growth of broilers

**DOI:** 10.1186/s40104-016-0099-3

**Published:** 2016-07-22

**Authors:** Yanli Liu, Lihui Zhi, Jing Shen, Shizhao Li, Junhu Yao, Xiaojun Yang

**Affiliations:** College of Animal Science and Technology, Northwest A&F University, Yangling, 712100 China; School of Mathematics and Computer Science, ShanXi Normal University, Linfen, 041000 China

**Keywords:** Broiler, Chromatin structure, Folic acid, Insulin-like growth factor 2, Methylation

## Abstract

**Background:**

Insulin-like factor 2 (*IGF2*) plays an important role in embryonic growth process by modulating intermediary metabolism and cell proliferation. Folic acid is involved in one carbon metabolism and contributes to DNA methylation which is related to gene expression. The purpose of this study was to explore whether folic acid could regulate *IGF2* expression via epigenetic mechanism and further promote embryonic growth of new-hatched broilers.

**Methods:**

In the present study, 360 fertile eggs were selected and randomly assigned to four treatments. On 11 embryonic day of incubation (E11), 0, 50, 100 and 150 μg folic acid were injected into eggs respectively. After hatched, growth performance of broilers were calculated. Hepatic *IGF2* expression, methylation level and chromatin structure of promoter region were analyzed.

**Results:**

Results have showed that *IGF2* expression was up-regulated in 150 μg folic acid group (*P* < 0.05) and other two dose of folic acid did not affect gene expression (*P* > 0.05). Meanwhile, methylation level of *IGF2* promoter were lower in 100 and 150 μg groups, which was consistent with lower expression of DNA methyltransferase 1 (*DNMT1*) (*P* < 0.05). What’s more, chromatin looseness of *IGF2* promoter was higher in 150 μg group than control group (*P* < 0.05). Further, birth weight (BW), liver and bursa index of new-hatched chickens in 150 μg folic acid group were higher than the other groups (*P* < 0.05). There were positive correlations between hepatic *IGF2* expression and BW and organs index (*P* < 0.05).

**Conclusion:**

In conclusion, our data have demonstrated that 150 μg folic acid injection on E11 could up-regulate *IGF2* expression by modulating DNA hypomethylation and improving chromatin accessibility in the gene promoter region, and ulteriorly facilitate embryonic growth and organ development of broilers.

**Electronic supplementary material:**

The online version of this article (doi:10.1186/s40104-016-0099-3) contains supplementary material, which is available to authorized users.

## Background

Insulin-like growth factor 2 (*IGF2*) has been generally studied in eutherian mammals and marsupial for the reason that *IGF2*/*H19* are imprinted and *IGF2* is associated with performance traits. However, genomic imprinting phenomenon has never been detected in chickens and *H19* gene is absent from the chicken sequence [1]. *IGF2* is located on chromosome 5 and displays a biallelic pattern of expression during chicken development [2]. Previous detailed reviews have reported the role of *IGFs* and provided ample evidence suggesting that *IGFs* were involved in DNA and protein synthesis, glucose and energy metabolism and lipid metabolism in poultry [3, 4]. Similarly to mammals, as an appropriate selection candidate gene, *IGF2* is also associated with economical traits of chicken such as muscle growth, body composition, embryonic growth and differentiation [2, 5].

The chicken is an important model organism in oviparous animals, and this species is well characterized in many biological aspects and bridges the evolutionary gap between mammals and other vertebrates [6]. Whereas, the energy and nutrients needed during embryonic development and growth of avian are stored in the egg. Nutritionally, the yolk, the egg richest fraction, is the primary source of nutrition. In *ovo* injection, a unique way of introducing nutrients into the incubating embryo, is used in poultry to improve nutritional level of eggs. Studies have indicated that in *ovo* injection of amino acids, vitamins as well as trace elements can enhance growth of the embryo and improve immune response during post-hatch development in chickens [7].

Folic acid is a key one carbon group involved in DNA, RNA and protein methylation as well as DNA synthesis and maintenance [8]. Epigenetics is to study the heritable changes in phenotype or gene expression without changes in the primary DNA sequence [9]. Many researches have reported the relationship between folic acid and DNA methylation [8, 10, 11]. IGF2 is mainly synthesized in liver and plays an important role in embryonic development of avian species via autocrine/paracrine mechanisms [12]. Moreover, transgenic and gene knockout mouse models have demonstrated that *IGF2* is required for tissue growth and fetal normal development [13, 14].

To sum up, on account of the importance of *IGF2* function, it will be valuable to study regulatory mechanism of *IGF2* expression, but to date correlational studies whether early nutrient supplementation might affect gene expression via epigenetic ways are scarce in poultry. Considering special characters during chicken growing and development, the present investigation was carried out to explore the effects of in *ovo* folic acid injection on hepatic *IGF2* expression and embryo development of broilers.

## Methods

### Animals experiment

In the study, 360 Cobb fertile eggs whose weight were among 65.7 ± 0.3 g were randomly assigned to four treatments (6 replicates/treatment, 15 birds/replicate). The experimental eggs were hatched in the microcomputer automatic incubator (Beijing LanTianJiao Electronic Technology Co., LTD, Beijing, China). All replicates of four treatments were arranged locally equalization as much as possible before hatching. Folic acid (Sigma, USA) was dissolved in sterile saline at 500, 1000 and 1500 μg/mL. Folic acid solution (0.1 mL) was injected into the yolk sac on E11 for three treatments respectively and another treatment was injected 0.1 mL saline as the control. The detailed injection methods were on the basis of previous report [15]. Injection time for folic acid was chosen according to the preliminary test where we have set set three times (E3, E7 and E11) for injection. Based on preliminary results (data not shown) and previous research [16], we select E11 as injection age in this study.

### Sample collection

During egg incubation, infertile and dead eggs were removed and recorded. After hatched, the fertilized hatchability was calculated by the formula: (the number of hatching birds/the number of fertilized eggs) * 100 %. One healthy bird was selected from each replicate to rapidly dissect whose weight was close to average value of the replicate. The liver, spleen, heart and bursa were removed and weighted immediately to calculate organ index. After weighting, all tissues were collected and frozen in liquid nitrogen. All samples were stored at −80 °C until analysis. All experimental protocol in the study was approved by the Animal Care and Use Committee of the College of Animal Science and Technology of the Northwest A&F University (Shaanxi, China).

### Genomic DNA and total RNA preparation

TIANamp Genomic DNA Kit (Tiangen, Beijing, China) was used to obtain hepatic genomic DNA. RNA extraction was performed according to the TRIzol Reagent protocol (Invitrogen, Carlsbad, USA). The concentration of DNA and RNA was determined by measuring the absorbance at 260 nm using a NanoDrop 2000c spectrophotometer (Thermo Fisher Scientific Inc, Delaware, USA). Finally, DNA was stored at −20 °C and prepared for bisulfite modification. RNA was diluted to a final concentration of 500 ng/μL and used to complete cDNA synthesis by Primer Script RT Reagent Kit (TaKaRa, Dalian, China). All cDNA samples were stored at −20 °C.

### Real-time quantitative PCR

The mRNA levels of *IGF2* and *DNMT1* in liver were quantified. The assay were carried out via the SYBR Premix Ex Taq kit (TaKaRa, Dalian, China) on the IQ5 (Bio-Rad, Hercules, USA). Reaction system of 20 μL contained the following: 1 μL cDNA, 1 μL each primer (10 pmol/μL), 10 μL SYBR Premix Ex Taq and 7 μL nuclease free water. Primers sequence were shown in Table 1. Protocols were set as follows: 95 °C for 5 min; followed by 40 cycles of 95 °C for 10 s, 60 °C for 30 s, and 72 °C for 30 s; with a final extension at 72 °C for 5 min. We ensured that data could be used for analysis when melting curve was specific and unimodal. Finally, the average cycle threshold (Ct) values after normalizing to β-action were used for quantification by the 2^−∆∆Ct^ method [17].

**Table 1:** Forwards (F) and reverse (R) primers of genes for RT-qPCR analysis

Genes	Accession number	Primer sequences, 5′to3′	Product size, bp
*β-actin*	L08165	F: ATTGTCCACCGCAAATGCTTC	113
		R: AAATAAAGCCATGCCAATCTCGTC	
*IGF2*	NM001030342	F: AGACCAGTGGGACGAAATAACA	131
		R: CACGCTCTGACTTGACGGAC	
*DNMT1*	NM206952	F: ACAGCCTTCGCCGATTACA	248
		R: CTCTCCACCTGCTCCACCAC	
*HAT1*	NM204207	F: AGAAGTTTGACTGTGTGGAGGC	152
		R: AGAATATGTGTGCAGCAGCATT	

### Bisulfite conversion and sequencing

The methylation of *IGF2* gene promoter was analyzed by bisulfite sequencing. For six replicates in each group, equal quantity of DNA from 2 samples was mixed as a DNA pool. Hence, three DNA pools from each group were performed by sodium bisulfite treatment using the EZ DNA Methylation Kit (Zymo Research, USA). The primers for bisulfite sequencing PCR (BSP) were as follows: forward TGG TTG TGT TGT AGA TTT TTT TTG T, reverse ACA CT AAA TTT CAC CTC CCA TTT T. They were designed by online Methprimer software (http://www.urogene.org/cgi-bin/methprimer/methprimer.cgi). Modified genomic DNA was served as the template for PCR amplification immediately and PCR products were gel purified using Gel Purification Kit (TaKaRa, Dalian, China). Then purified DNA were cloned into the pMD19-T vector (TaKaRa, Dalian, China) and used for transformation of competent *Escherichia coli*. Later, cells were plated on LB ager and identified by blue-white selection. Detailed methods above were under previous research [18]. Positive clones for each subject were randomly selected and grown overnight into LB broth and then LB broth were collected for sequencing (Sangon, Shanghai, China). The final sequence results were analyzed by online QUMA software (http://quma.cdb.riken.jp/).

### Chromatin looseness by DNase-qPCR assay

Nuclei from the liver tissue were isolated using Nuclear Extraction Kit (Solarbio, Beijing, China). DNase I digestion and qPCR assays were carried out as described by the methods [19, 20]. Briefly, nuclei were digested with DNase I (Thermo, Beijing, China). Then protein digestion was performed by proteinase K. Finally, DNA in the reaction mixture was purified using QIAquick PCR Purification Kit (Qiagen, Germany). Purified DNA was used for PCR. Reaction system was similar to the qPCR assay. The primer had following sequence: forward CAG GTG GTG CTG CGA TGA C, reverse CGG AGA TGG AGC CGA AGC. The cycle programs were performed as follows: an initial step at 95 °C for 15 min; then 10 cycles of 94 °C for 15 s, 70 °C for 30 s and 72 °C for 45 s; followed by 35 cycles of 94 °C for 15 s, 60 °C for 30 s and 72 °C for 45 s. Data were analyzed as previously reported [20].

### Statistical analysis

Experimental data were analyzed by one way ANOVA and regression analysis using SPSS 20.0 (SPSS Inc., Chicago, IL, USA). Correlation analysis were conducted by Pearson correlation. A probability value of *P* < 0.05 was considered to be statistically significant and the notable differences between groups were identified by Duncan’s multiple comparisons test. The methylation levels of each CpG site were tested by Fisher’s exact test and the total methylation was tested by Mann-Whitney U-test according to the online QUMA software.

## Results

### Performance of new-hatched broilers

The fertilized hatchability of four groups are orderly 79.2, 79.3, 90.9 and 89.6 % (data not shown). Based on the Chi-square test, 100 and 150 μg folic acid improved the hatchability compared with the control group (*P* < 0.05). As shown in Table 2, BW, liver and bursa index of chickens in 150 μg folic acid group were markedly higher than the other groups. However, folic acid had no effects on spleen and heart index.

**Table 2:** Effects of folic acid injected at E11 on organ index and birth weight of broilers

Item	Folic acid, μg				SEM	*P*-value
	0	50	100	150		
^1^BW, g	43.00^bc^	42.25^c^	44.00^b^	45.67^a^	0.432	0.005
^2^Spleen index, 10^−2^	0.036	0.029	0.033	0.045	0.002	0.065
Heart index, 10^−2^	0.603	0.485	0.633	0.693	0.030	0.054
Liver index, 10^−2^	1.63^b^	1.33^b^	1.58^b^	2.21^a^	0.103	0.001
Bursa index, 10^−2^	0.082^b^	0.063^c^	0.073^bc^	0.100^a^	0.005	0.004

^1^BW = birth weight of new-hatched broilers; Organ index = organ weight (g)/birth weight (g)

^2^In the same line, values with different small letter superscripts mean significant difference (*P* < 0.05)

### *IGF2* expression

**Fig. 1:**
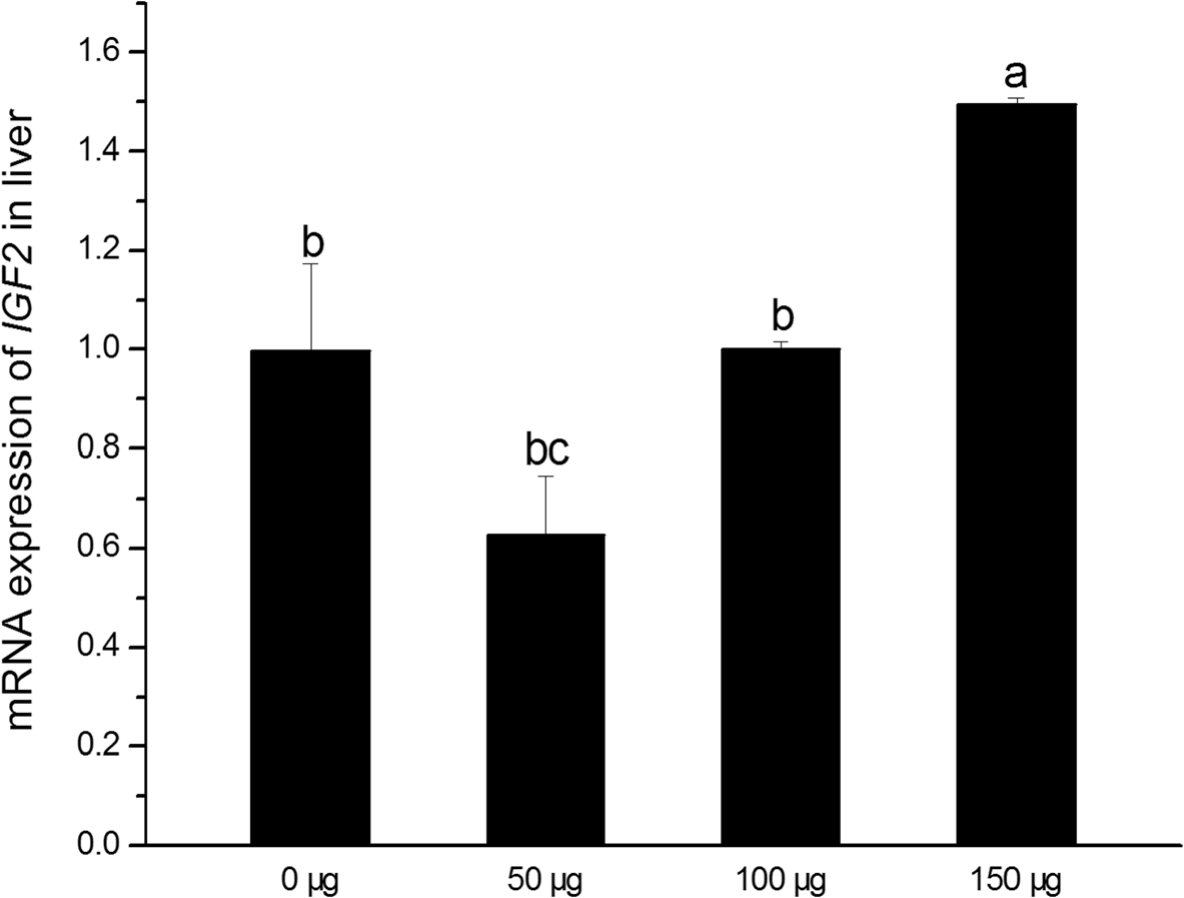
Effects of in *ovo* folic acid injection on *IGF2* mRNA expression in the liver of new-born chickens. Numerical values (0, 50, 100, 150 μg) in X-axis mean the amount of folic acid which was injected into eggs. Data were presented as means ± SEM (*n* = 6). Bars with different letters are significantly different (*P* < 0.05). The *P* values for linear and quadratic regression analysis were 0.324 and 0.225, respectively

Hepatic *IGF2* expression of birth chicken was shown in Fig. 1. Results indicated that injecting 150 μg folic acid on E11 up-regulated *IGF2* expression (*P* < 0.05), but there were no significant differences between control and lower dose folic acid groups. No curve fitting methods was found by regression analysis using SPSS statistical software (*P* > 0.05).

### Methylation levels of *IGF2* promoter region

**Fig. 2:**
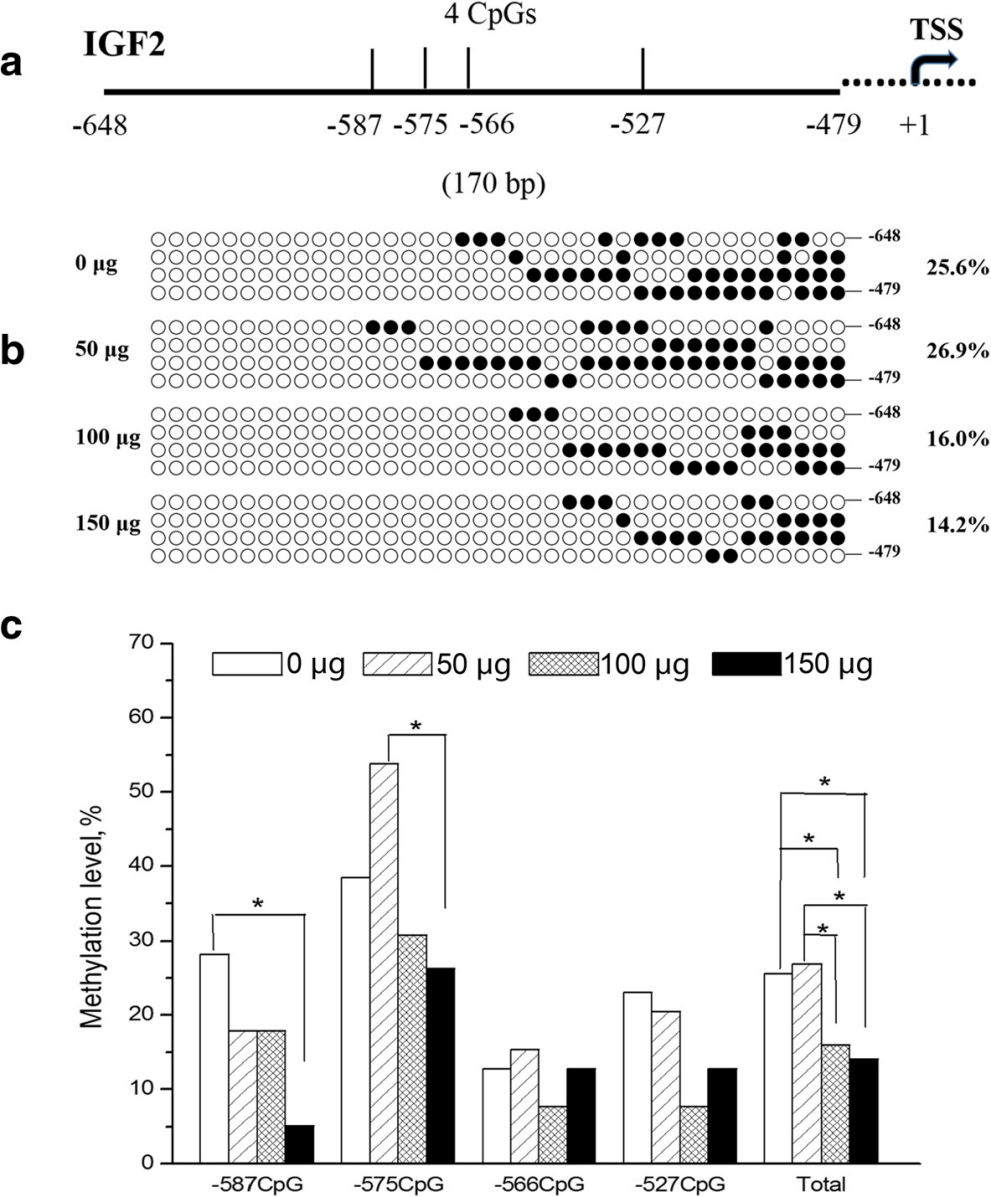
Methylation analysis of the *IGF2* promoter region in the liver. **a** Map of CpG dinucleotide in *IGF2* promoter regions are presented by the ratable line. Numbers under the line are the positions relative to the transcription start site (TSS). **b** The methylation patterns of each CpG site were shown as maps of empty and full circles. Empty circles showed unmethylated CpGs and full circles methylated CpGs. Each vertical line is made up of four circles and represent one bacterial clone. **c** The total methylation and each CpG site methylation level of promoter region were calculated by analyzing the 39 clones in total. The asterisk (*) indicated statistically significant differences (*P* < 0.05). The *P* values for linear and quadratic regression analysis were > 0.05

The methylation levels of *IGF2* promoter were shown in Fig. 2. The methylation of −566 and −527 CpG sites were not affected by the folic acid. However methylation level of −587 CpG site decreased significantly in 150 μg folic acid group when compared with the control. As for −575 CpG site, methylation level in 150 μg folic acid group was lower than that in 50 μg folic acid group. Total methylation level of *IGF2* promoter were lower in 100 and 150 μg folic acid groups than the control and 50 μg folic acid groups (*P* < 0.05). No curve fitting methods was found by regression analysis using SPSS statistical software (shown in Additional file 1) for total and single site methylation level (*P* > 0.05).

### Chromatin looseness of *IGF2* promoter region

**Fig. 3:**
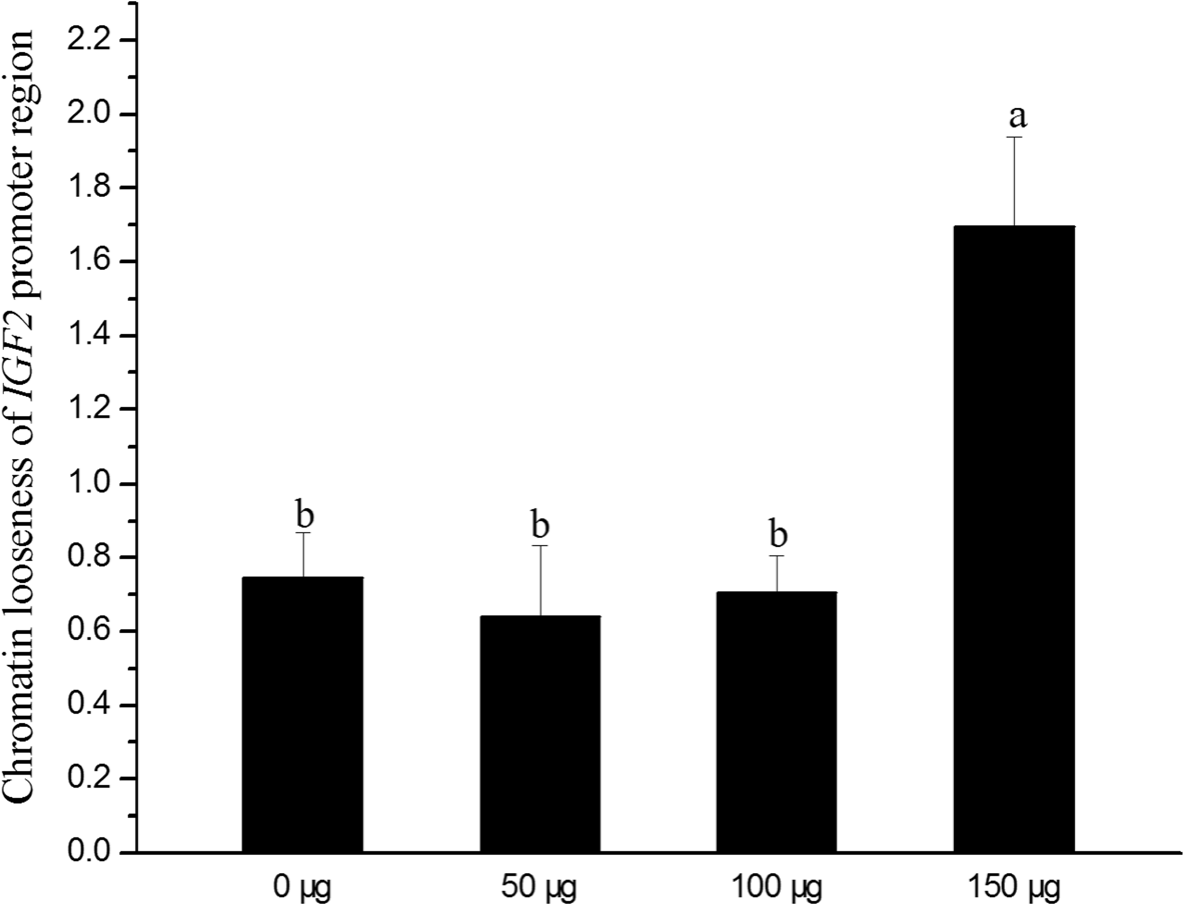
Effects of in *ovo* folic acid injection on chromatin looseness of *IGF2* promoter region in the liver. Numerical values (0, 50, 100, 150 μg) in X-axis mean the amount of folic acid which was injected into eggs. Bars with different letters are significantly different (*P* < 0.05)

As depicted in Fig. 3, the promoter region revealed higher chromatin looseness in 150 μg folic acid group after DNase I digestion when compared with the other groups (*P* < 0.05), while 50 or 100 μg folic acid didn’t affect chromatin looseness of *IGF2* promoter region.

### *DNMT1* expression

**Fig. 4:**
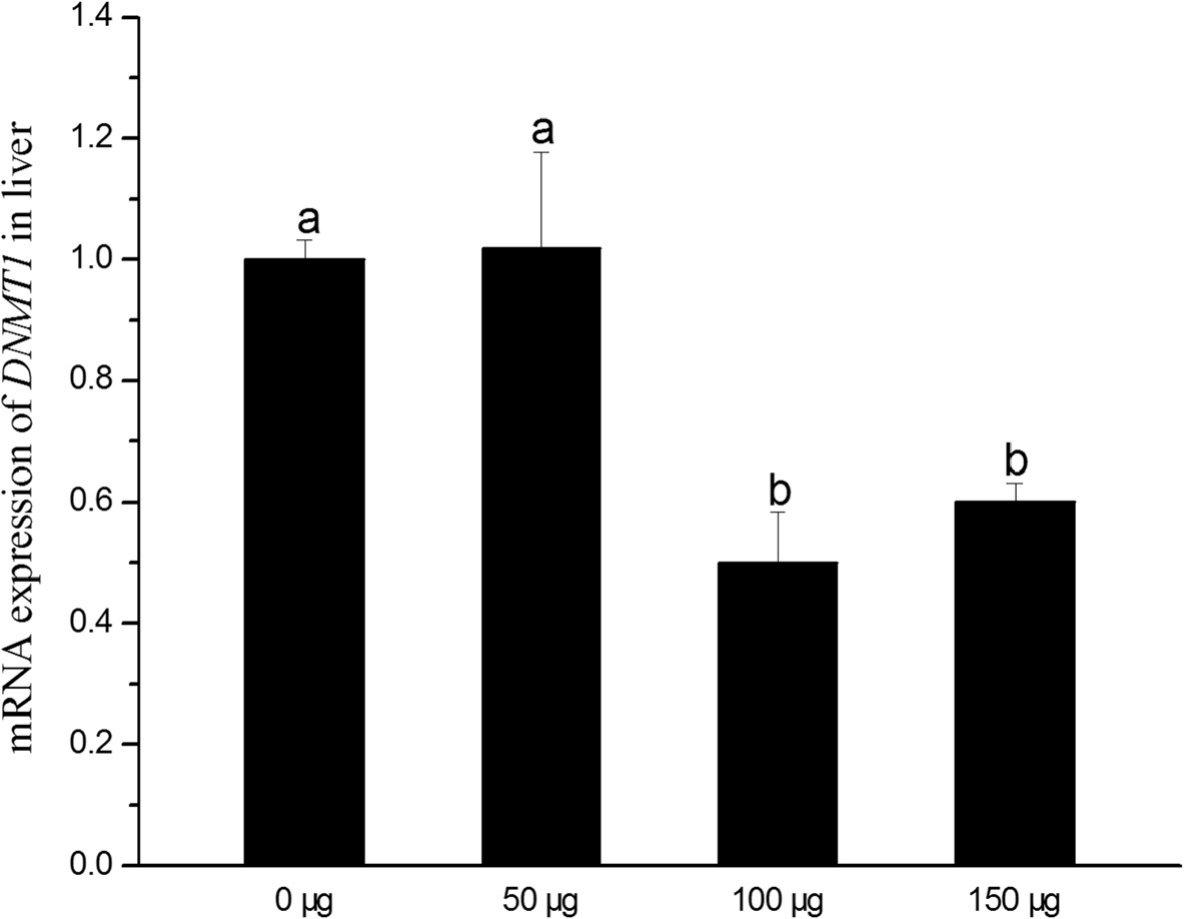
Effects of in *ovo* folic acid injection on hepatic *DNMT1* expression in the new-born chickens. Numerical values (0, 50, 100, 150 μg) in X-axis mean the amount of folic acid which was injected into eggs. Data were presented as means ± SEM (*n* = 6). Bars with different letters are significantly different (*P* < 0.05). The *P* values for linear and quadratic regression analysis were 0.175 and 0.558, respectively

As shown in Fig. 4, *DNMT1* expression was down-regulated in 100 and 150 μg folic acid groups (*P* < 0.05). Compared with control group, 50 μg folic acid injection didn’t affect *DNMT1* expression of liver. No curve fitting methods was found by regression analysis using SPSS statistical software (*P* > 0.05).

### Transcription factor binding sites prediction

**Fig. 5:**
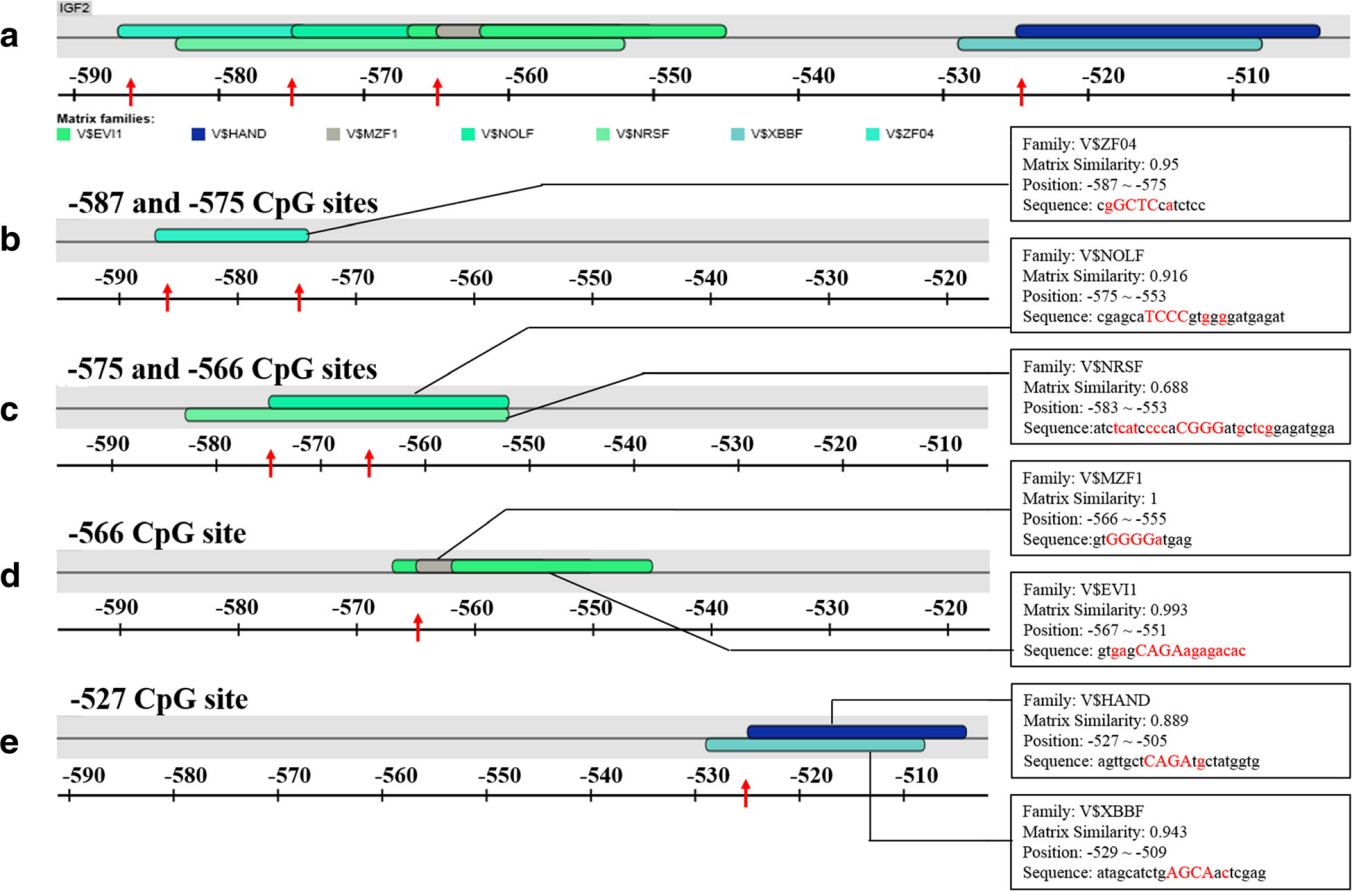
Transcription factor binding site (TFBS) prediction in the *IGF2* promoter regions. **a** Total predicted transcription factors involving the four CpG sites in the promoter of *IGF2* gene. The red arrows represent the distribution of the CpG loci. **b** The partial predicted transcription factors related to −587 and −575 CpG sites and the same as below. **c**, **d** and **e** are correlated with −575 and −566 CpG sites, −566 CpG site and −527 CpG site respectively. Contents in the boxes describe detailed information of transcription factors which might combine the region containing the CpG sites

In order to speculate the relationship between *IGF2* mRNA expression and the methylation of 4 CpGs examined in the *IGF2* promoter regions, the sequence including the 4 CpGs was submitted into the online software (http://www.genomatix.de) to obtain the latent transcription factors bound at the 4 CpG sites. Results predicted are shown in Fig. 5. In line with expectations, there were 7 potential transcription factors found in the predictive sequence, and introductions of these transcription factors were presented in Fig. 5b, c, d and e.

### Correlations between hepatic *IGF2* and BW and organ index

As shown in Table 3, results indicated that BW of chickens and organ index was positively related to *IGF2* expression level of the liver (*P* < 0.01). What’s more, BW showed a significantly positive correlation with the organs index (*P* < 0.01).

**Table 3:** Correlation between hepatic *IGF2* expression and birth weight and organ index

Item	*IGF2*	BW	Spleen	Heart	Liver	Bursa
*IGF2*	1					
BW	0.945^a^	1				
Spleen	0.898^a^	0.872^a^	1			
Heart	0.932^a^	0.894^a^	/	1		
Liver	0.954^a^	0.928^a^	/	/	1	
Bursa	0.942^a^	0.856^a^	/	/	/	1

^a^shows that there is a significant correlation between two indices at the 0.01 level (two- tailed)

## Discussion

Mechanisms of epigenetic regulation contain DNA methylation, histone modification, chromatin remodeling in mammals, which modulate chromatin structure and contribute to regulation of molecular processes including transcription and repair [21]. The previous review has proposed that DNA methylation in the promoters or other regulatory regions might prevent stable binding of regulatory activator proteins to that sequence, thereby preventing gene expression [21]. Another mechanism is mediated by DNA-binding proteins which contain methylated DNA-binding domains [22]. The methylated CpG dinucleotide are recognized and bound by these methylated DNA-binding proteins, then the proteins in turn interact with or recruit other transcriptional silencing complexes which work to form a tight chromatin structure at the relevant gene [23]. It is a big challenge for researchers to understand epigenetic mechanisms of gene regulation absolutely in biological progress.

Many studies suggested that *IGF2* is indeed more influential than *IGF1* for embryo and organ growth in the embryonic development of birds [24–26]. Our data showed that hepatic *IGF2* expression was up-regulated by injecting 150 μg folic acid on E11 during incubation period, while 50 and 100 μg folic acid might be too less to reach the same effect. Meanwhile, BW, liver and bursa index of new-hatched chickens in 150 μg folic acid group increased when compared with the control. Previous study reported that there were correlations between body weight of chick embryo and plasma IGFs level [27]. The liver, as a center of metabolism, was considered to be the major source of blood IGF2 circulation which exerted endocrine functions [12]. It’s well apparent that hepatic *IGF2* expression is important for the growth and development of broilers embryos. Previous study indicated that body and liver weight were related to hepatic *IGFs* gene expression in ducks [24].

In order to further demonstrate whether the methylation of *IGF2* promoter was served as the regulatory factor for gene expression, we detected the methylation status of *IGF2* gene promoter region. What is more intriguing was that methylation level of *IGF2* promoter region was lower in 150 μg folic acid group, which was consistent with the result of *IGF2* overexpression. DNA methylation affected gene expression by disturbing the binding of specific transcription factors [28]. Whereupon it could be legitimately extrapolated that hypomethylation of *IGF2* promoter might activate gene transcription in virtue of attracting the co-localized transcription factors, subsequently up-regulating *IGF2* expression. Just as the prediction, 7 various potential transcription factors were located abundantly at four CpGs in the promoter region.

Although folic acid’s role in one carbon metabolism is related to increasing DNA methylation, from other aspects it is not difficult to understand the phenomenon in the study that folic acid decreased total methylation level of gene promoter region. Firstly, some other nutrients are involved in one carbon flux to ensure homocysteine remethylation, S adenosyl methionine (SAM) formation and DNA methylation such as choline, betaine and other B vitamins [11]. Therefore methylation status is not only depended on the folic acid. Secondly, methionine is the substrate for SAM, which is the methyl donor in transmethylation reaction of DNA methylation [29]. The conversion of homocysteine to methionine benefits from the methyl group donated by 5-Methyl-THF, which finally turns into tetrahydrofolate (THF) in this bidirectional reaction. However folic acid is originally first reduced to dihydrofolate (DHF) and then to THF by DHF reductase, thus the level of THF produced from folic acid might suppress homocysteine remethylation reaction. Besides, homocysteine remethylation is the only known reaction involving 5-methyl THF whose synthesis is in a unidirectional reaction catalyzed by methylenetetrahydrofolate reductase (MTHFR). The production of 5-methyl THF by MTHFR is an important and regulatory step in one carbon metabolism cycle. But SAM is a potent inhibitor of MTHFR, and folic acid may disturb regulation of one carbon metabolism by interfering with the inhibitory effect of SAM on MTHFR activity [30].

The mechanisms associated with effects of folic acid on DNA methylation are complex and not fully understood. The previous study found that using of folic acid before and during pregnancy was connected with lower methylation levels at DNA sequences regulating *IGF2* expression [31], which suggested that folic acid might lower methylation level in site-specific DNA of the gene. Another research indicated that increased serum folate among smokers was associated with decreased methylation of five detected genes [32]. What’s more, the lowest methylation level at the second site of the *PPARγ* promoter was examined in cells exposed to 4 mg/L of folate [33]. These researches all conformed to the effects of folic acid on DNA hypomethylation of *IGF2* promoter region in our study.

One possible mechanism that caused changes in methylation level as well as chromatin structure was enzymes activity. *DNMT1* plays a vital part in the maintenance of methylation especially in tissues of adults after fertilization [34], and unnatural expression of *IGF2* was found in *DNMT1* knock-out mice [35]. In present study, we detected lower expression of hepatic *DNMT1* in 100 and 150 μg folic acid groups which were consistent with the results of DNA hypomethylation. Hypomethylation of a conserved single CpG in *DNMT1* had a positive correlation with gene expression of *DNMT1* [36]. The previous study showed that choline deficiency induced the hypomethylation of regulatory CpG in the *DNMT1* gene, leading to overexpression of *DNMT1* and the final higher methylation level in global and gene-specific DNA [18]. Thus, taken together, our data obviously pointed to the relationship that in *ovo* folic acid injection on E11 caused decline of *DNMT1* expression followed by hypomethylation in *IGF2* promoter regions leading to an acceleration of *IGF2* expression. Likely, the former may be due to the hypermethylation of the CpG in *DNMT1* regulatory region induced by folic acid.

The phenomenon was doubtful that low methylation level of *IGF2* promoter and *DNMT1* down-regulation in 100 μg folic acid group didn’t result in *IGF2* overexpression. We examined the chromatin structure of *IGF2* promoter in order to illuminate the doubt. Our data showed that another contributing factor for the detected hepatic *IGF2* overexpression was chromatin looseness of *IGF2* promoter. Because 150 μg folic acid injection improved chromatin looseness in the study, but this result was not found in 100 μg folic acid group. Gene expression could be induced by local chromatin structures which regulate transcription factor binding activities [37]. Chromatin is a DNA-protein complex, and protein components are core histones. Hence, it might be probable that chromatin of *IGF2* promoter region was changed into loose status to facilitate gene transcription. So the doubt mentioned above could be clarified. The possible explanation is that *IGF2* transcription might depend on both methylation and chromatin structure of *IGF2* promoter regions.

In the study, lower level of folic acid didn’t affect hepatic *IGF2* expression. It was likely that the dose of 50 and 100 μg folic acid might be not enough to change the methylation and chromatin structure at the same time. Automatically embryonic growth and organ development of new-hatched chicken couldn’t gain corresponding improvement. Even so, the internal causal relationship between methylation status and chromatin structure of *IGF2* promoter regions is unclear.

## Conclusion

In conclusion, the present study has demonstrated that the effects of in *ovo* folic acid injection on hepatic *IGF2* expression and embryo growth of chickens. Our results likely have general implications that embryo growth and organ development of chicken have a positive correlation with hepatic *IGF2* expression. 150 μg folic acid injected on E11 might up-regulate *IGF2* expression by decreasing methylation and improving chromatin accessibility of gene promoter, which offers new insights into the field of nutri-epigenetics. But internal relationship between DNA methylation and chromatin structure affected by folic acid is expected for further research.

## Additional file

Additional file 1: The summary of regression analysis for methylation data. (DOC 42 kb)
