# Supplementary material for: Effect of in ovo folic acid injection on hepatic IGF2 expression and embryo growth of broilers
Source: J Anim Sci Biotechnol. 2016 Jul 22;7:40. doi: 10.1186/s40104-016-0099-3 (PMC4957392; doi:10.1186/s40104-016-0099-3)
Supplement: Additional file 1: — The summary of regression analysis for methylation data. (DOC 42 kb) [file 40104_2016_99_MOESM1_ESM.doc]

The summary of regression analysis for methylation data

| Types of regression curve | Methylation sites | *P*-value |
| --- | --- | --- |
| Linear | Total | 0.103 |
|  | -587 CpG | 0.064 |
|  | -575 CpG | 0.362 |
|  | -566 CpG | 0.692 |
|  | -527 CpG | 0.202 |
| Quadratic | Total | 0.419 |
|  | -587 CpG | 0.315 |
|  | -575 CpG | 0.608 |
|  | -566 CpG | 0.925 |
|  | -527 CpG | 0.513 |
| Compound | Total | 0.088 |
|  | -587 CpG | 0.099 |
|  | -575 CpG | 0.292 |
|  | -566 CpG | 0.700 |
|  | -527 CpG | 0.291 |
| Growth | Total | 0.088 |
|  | -587 CpG | 0.099 |
|  | -575 CpG | 0.292 |
|  | -566 CpG | 0.700 |
|  | -527 CpG | 0.291 |
| Exponential | Total | 0.088 |
|  | -587 CpG | 0.099 |
|  | -575 CpG | 0.292 |
|  | -566 CpG | 0.700 |
|  | -527 CpG | 0.291 |
